# The Association Between Laterality and Stroke Severity: A Cross‐Sectional Study

**DOI:** 10.1002/hsr2.72102

**Published:** 2026-03-22

**Authors:** Imen Ezzouch, Riadh Dahmen, Kaouther Mejri, Liwa Masmoudi, Saoussen Daoued, Dalia Elleuch, Sana Ben Amor, Mariem Dammak, Chokri Mhiri, Haitham Jahrami, Achraf Ammar, Khaled Trabelsi, Mohamed Jarraya

**Affiliations:** ^1^ Higher School of Health Sciences and Techniques University of Sfax Sfax Tunisia; ^2^ High Institute of Sport and Physical Education of Sfax University of Sfax Sfax Tunisia; ^3^ Research Laboratory, Education, Motricity, Sport and Health (EM2S), LR15JS01 University of Sfax Sfax Tunisia; ^4^ Laboratory of Neurogenetics, Parkinson's Disease and Cerebrovascular Disease, LR12SP19, Habib Bourguiba University Hospital University of Sfax Sfax Tunisia; ^5^ Faculty of Medicine of Sfax University of Sfax Sfax Tunisia; ^6^ Neurology Department of Sahloul Hospital University of Sousse Sousse Tunisia; ^7^ Government Hospitals Manama Bahrain; ^8^ Department of Psychiatry, College of Medicine and Medical Sciences Arabian Gulf University Manama Bahrain; ^9^ Department of Training and Movement Science, Institute of Sport Science Johannes Gutenberg‐University Mainz Mainz Germany; ^10^ Research Laboratory, Molecular Bases of Human Pathology, LR19ES13, Faculty of Medicine of Sfax University of Sfax Sfax Tunisia; ^11^ Nutrition and Food Technology Department, School of Agriculture The University of Jordan Amman Jordan; ^12^ Department of Movement Sciences and Sports Training, School of Sport Science The University of Jordan Amman Jordan; ^13^ Research Laboratory: Education, Motricity, Sport and Health, EM2S, LR19JS01, High Institute of Sport and Physical Education of Sfax University of Sfax Sfax Tunisia

**Keywords:** footedness, functional laterality, handedness, ocular preference, stroke

## Abstract

**Background and Aim:**

Handedness is a manifestation of cerebral lateralization that has been implicated in various neurological and neurodevelopmental conditions. Despite extensive research, the relationship between laterality and stroke outcomes remains underexplored. This study investigates the association between laterality (handedness, footedness, and ocular preference) and stroke severity.

**Methods:**

A cross‐sectional study of 98 stroke patients from Tunisian hospitals assessed laterality (handedness, footedness, ocular preference) and NIHSS severity. Laterality was evaluated using validated questionnaires for manual, foot, and ocular preferences. Statistical analyses included nonparametric tests to examine the correlation between different aspects of laterality, the relationship between laterality (handedness, footedness, and ocular preference) and both stroke risk factors and stroke severity, as well as the correlation between stroke risk factors and severity, with a particular emphasis on the indirect impact of laterality.

**Results:**

The majority of participants were right‐handed (66.3%), while 10.2% were left‐handed and 38.8% demonstrated crossed laterality. No significant association was found between laterality and stroke severity (*p* > 0.05). Left‐handedness was more prevalent among dyslipidemic patients, a factor significantly associated with stroke severity (OR = 5.6, *p* = 0.033).

**Conclusion:**

The findings suggest that while laterality does not directly influence stroke severity, it may indirectly affect outcomes by modulating stroke risk factors. Therefore, laterality plays a crucial role in stroke, primarily through its impact on risk factors rather than directly determining severity.

**Abbreviations**
CI: confidence intervals
HBP: high blood pressure
LF: left‐footed
LH: left‐handed
LI: Laterality index
NIHSS: National Institutes of Health Stroke Scale
OR: odds ratio
RF: right‐footed
RH: right‐handed

## 1 Introduction

The influence of handedness on cerebral structure, function, and health outcomes has long been a focal point of neurobiological research [1, 2]. Recent studies have demonstrated significant asymmetries in cortical thickness within language‐related and motor control regions in both right‐handers and left‐handers [3, 4, 5, 6, 7, 8].

Handedness, the preferential use of one hand over the other, is a manifestation of brain lateralization, which is a fundamental aspect of neural organization that reflects the functional and structural asymmetry between the left and right hemispheres of the brain [9, 10]. While ~90% of the global population is right‐handed, the remaining 10% exhibit left‐handedness or mixed‐handedness, presenting a unique neurobiological profile characterized by more symmetrical brain organization [7]. This atypical lateralization has been linked to distinct cognitive and motor abilities, as well as differential susceptibility to neurological and psychiatric disorders [11, 12, 13, 14, 15].

Stroke, a leading cause of mortality and long‐term disability worldwide, represents a major public health challenge [16]. According to the Global Burden of Disease, stroke accounted for 12.2% of total deaths globally in 2019, with an estimated 101 million prevalent cases and 143 million disability‐adjusted life years lost [17]. The medical, social, and economic consequences of stroke are profound, underscoring the urgent need for personalized treatment strategies to improve patient outcomes [18, 19]. Given the variability in stroke severity and recovery, understanding the role of individual factors, such as laterality, is essential for refining therapeutic approaches.

Brain laterality, characterized by left‐hemisphere dominance for language and fine motor control and right‐hemisphere involvement in spatial processing and emotional regulation, is observed across species, with left‐handed individuals often showing more balanced language distribution across hemispheres, potentially influencing stroke outcomes [9, 10, 20, 21]. In fact, lateralization has been hypothesized to influence stroke outcomes, with some studies suggesting that left‐handed individuals may recover more effectively due to enhanced interhemispheric connectivity [7].

The relationship between handedness and initial stroke severity is further complicated by the phenomenon of neuroplasticity, which is a cornerstone of acute stroke severity, enabling the brain to compensate for lost functions through mechanisms such as synaptic reorganization and the recruitment of alternative neural pathways [22, 23]. Recent evidence suggests that left‐handed individuals, with their more symmetrical brain organization, may demonstrate greater neural adaptability following stroke, potentially leading to improved severity upon admission [24]. However, the clinical implications of this phenomenon remain unclear, necessitating further investigation.

Beyond its potential impact on stroke recovery, handedness has been associated with a range of health conditions that may indirectly influence stroke risk and severity. Left‐handed individuals exhibit higher rates of comorbidities such as cardiovascular disease, diabetes, and migraines, along with a greater prevalence of dyslipidemia, a known risk factor for stroke [25, 26]. These findings suggest that left‐handed individuals may face a greater health burden, though the underlying mechanisms remain unknown. Furthermore, an association between handedness and carotid artery characteristics, which are indirectly linked to stroke risk, has been demonstrated [27, 28].

The role of footedness and ocular preference, which may provide additional insights into brain lateralization, has been largely overlooked in the context of stroke research. Footedness, in particular, is a more reliable predictor of emotional lateralization than handedness, suggesting that foot preference is linked to brain organization [29, 30]. Moreover, the complex interplay between hemispheric specializations for manual preference, ocular dominance, and language processing underscores the need for a comprehensive approach to studying laterality and its impact on stroke severity [31].

In rare instances, handedness may reflect disturbances in cerebral development and be linked to associated health conditions. For example, a high prevalence of left‐handedness has been reported in children with athetoid cerebral palsy [32]. Most children with cerebral palsy are left‐handed [33]. Additionally, children with cerebral diplegia have a high likelihood of being left‐handed [34]. These findings suggest that left‐handedness may be linked to neurodevelopmental conditions, which could have implications for stroke risk and initial stroke severity [12].

Given the established relationship between laterality and neurological pathology, together with the high prevalence and incidence of stroke in Tunisia [35, 36], this study aims to examine the association between stroke severity and laterality, including handedness, footedness, and ocular preference. We hypothesize that non‐right‐handedness would be associated with higher stroke severity; thus, we aim to analyze which characteristic (left‐handers, right‐handers, mixed handers, crossed laterality) is linked to stroke severity. The findings of this study aim to help physiotherapists and physical activity trainers adapt their rehabilitation protocols and physical training programs based on the specific needs and laterality of their patients.

## 2 Materials and Methods

### 2.1 Study Design and Participants

This cross‐sectional study included 98 patients (33 females and 65 males) who had experienced ischemic or hemorrhagic stroke. Participants were recruited from the neurology departments of Habib Bourguiba University Hospital in Sfax and Sahloul Hospital in Sousse. The sample size (*n* = 98) was determined by the availability of eligible participants meeting the inclusion criteria during the recruitment period (January 2022–December 2023). Ethical approval was obtained from the Ethics Committee (Reference No. 452/2022), and verbal informed consent was obtained from patients in accordance with the ethical standards of the revised 2024 Declaration of Helsinki. STROBE Checklist of items that should be included in reports of cross‐sectional studies is provided in Supporting File S1.

#### 2.1.1 Inclusion Criteria

We included all patients who had suffered from ischemic or hemorrhagic stroke at the acute phase, resulting in a motor deficit on the right or left side (right and left hemiplegia) or without any muscular deficit (coordination and balance disorders), who had neither a comprehension nor a communication problem, and who were able to answer the questionnaires.

#### 2.1.2 Exclusion Criteria

Patients with stroke with communication, comprehension, or memory problems were not included in our study. Cognitive function was assessed using the Mini‐Mental State Examination (MMSE) [37]. To account for the influence of education on cognitive performance, the threshold for cognitive impairment was set at < 27 for participants with an average to high educational level and < 24 for those with a low educational level or language difficulties. Participants scoring below these respective thresholds were classified as cognitively impaired and excluded from further analyses, in accordance with established age‐ and education‐adjusted norms [38, 39, 40]. In a sensitivity analysis, classifications were cross‐validated using the Montreal Cognitive Assessment (MoCA), which offers greater sensitivity for detecting poststroke cognitive deficits [41, 42].

#### 2.1.3 Data Collection

##### 2.1.3.1 Stroke Severity Assessment

Stroke severity was evaluated using the National Institutes of Health Stroke Scale (NIHSS) [43], a validated and widely used tool for assessing initial neurological deficit. The NIHSS score, which ranges from 0 (*no stroke symptoms*) to 42 (*severe stroke*), was calculated by neurologists upon admission. The scale evaluates various domains, including level of consciousness, motor function, language, and sensory deficits.

##### 2.1.3.2 Laterality Assessment

a. Handedness: Hand preference was assessed using the Dellatolas Handedness Inventory [44], which includes 10 tasks such as writing, brushing teeth, throwing a ball, and using utensils. Participants were asked to indicate their preferred hand for each task (right, left, or no preference).
b. Footedness: Foot preference was evaluated using the Dahmen Footedness Inventory [45], which assesses tasks such as kicking a ball, stepping on an object, and balancing on one foot.
c. Ocular Dominance: Ocular preference was determined by asking participants about their preferred eye for specific tasks, such as looking through a telescope or aiming at a target.

Laterality Index (LI) was assessed using the Delatolas Laterality Inventory [44]. The total score was calculated according to the standardized scoring procedure. A score of 0 was classified as strong right‐handedness. Scores ranging from 1 to 6 were categorized as mixed right‐handedness, scores between 7 and 15 as mixed left‐handedness, and scores between 17 and 20 as strong left‐handedness. This classification system provided a clear framework for assessing the degree and direction of handedness in the study population.

##### 2.1.3.3 Demographic and Clinical Data

Additional data collected included age, sex, medical history (e.g., hypertension, diabetes, dyslipidemia), lifestyle factors (e.g., smoking, alcohol consumption), and stroke type (ischemic or hemorrhagic).

##### 2.1.3.4 Bias Considerations

Potential selection bias was mitigated by consecutively recruiting all eligible patients from both hospital sites. However, results may not generalize to stroke patients with communication impairments (excluded per study criteria) or nonhospitalized populations.

Ethical approval was obtained from the Habib Bourguiba University Hospital Ethics Committee (Reference No. 452/2022), and written informed consent was obtained from all patients in accordance with the ethical standards of the revised 2024 Declaration of Helsinki.

### 2.2 Statistical Analysis

We used IBM SPSS Statistics version 25.0 to perform the statistical analyses. Categorical variables were summarized by their frequencies and percentages. Fisher's exact test for 2 × 2 contingency tables was employed to examine associations between categorical variables, as recommended for small sample sizes. Shapiro–Wilk tests confirmed the non‐normal distribution of continuous variables, justifying the use of Spearman's rank‐order correlations to assess relationships between continuous variables. Effect sizes for 2 × 2 tables were reported as odds ratios (OR) with 95% confidence intervals. All tests were two‐tailed, and *p* < 0.05 was used to determine statistical significance. The number of samples used in the analyses ranged from 73 to 98.

## 3 Results

### 3.1 Participant Characteristics

A total of 98 patients (mean age: 61.81 ± 11.97 years) were included in this cross‐sectional study, with a male predominance (sex ratio M/F = 2.06). The majority of participants were over 60 years old (66.3%). Ischemic stroke was significantly more prevalent (84.7%, *n* = 83) compared to hemorrhagic stroke (15.3%, *n* = 15). Common comorbidities included hypertension (54.1%), diabetes (38.8%), cardiac disease (16.3%), and dyslipidemia (13.3%). Lifestyle habits revealed that 28.6% were cigarette smokers and 7.1% were alcohol drinkers (Table 1).

**Table 1:** Participant demographic and clinical characteristics.

Characteristics	Values
Total number of patients	*n* = 98
Mean age ± standard deviation	61.81 ± 11.97
Sex ratio (M/F)	2.06
Age > 60 years	66. 3%
Stroke type	
Ischemic	84.7% (*n* = 83)
Hemorrhagic	15.3% (*n* = 15)
Comorbidities	
High blood pressure	54.1
Diabetes	38.8%
Cardiac disease	16.3%
Dyslipidemia	13.3
Lifestyle habits	
Cigarette smokers	28.6%
Alcohol consumers	7.1%

### 3.2 Laterality Profile

The LI was calculated to assess manual, foot, and ocular preferences. The majority of patients were right‐handed (66.3%, *n* = 65), while 2% (*n* = 2) were left‐handed and 23.5% (*n* = 23) were mixed right‐handed. Left‐footedness was observed in 12.24% (*n* = 12) of patients, with 7.14% (*n* = 7) exhibiting both left‐handedness and left‐footedness. Ocular preference was predominantly right‐sided (69.4% monocular, 71.4% binocular). Cross‐laterality was observed in 38.8% (*n* = 38) of patients. A significant association was observed between manual and foot preferences (*p* < 0.01) (Table 2).

**Table 2:** Laterality profile.

Laterality type	*N*	%	Reference value	References
Manual preference				
Right‐handed	65	66.3%	90%	Papadatou‐Pastou et al. [7]
Left‐handed	2	2.0%	10%–11%	
Mixed right‐handed	23	23.5%		
Mixed left‐handed	8	8.2%	9.33%	
Foot preference				
Right‐footed	39	39.8%		
Left‐footed	3	3.1%	12%	Packcheiser et al. [46]
Mixed right‐footed	47	48.0%		
			20%–24%	
Mixed left‐footed	9	9.2%		
Monocular preference				
Right monocular	68	69.4%		Bourassa et al. [47]
Left monocular	12	12.2%	28%–36%	
No preference	18	18.4%		
Binocular preference				
Right binocular	70	71.4%		
Left binocular	18	18.4%		
No preference	10	10.2%		

Specifically, manual preference was strongly correlated with foot preference (*p* < 0.01). Additionally, ocular and binocular preferences demonstrated significant associations with both manual and foot laterality (*p* < 0.01) (Table 3).

**Table 3:** Associations between laterality types.

Ocular preference	Manual					Foot			
		RH	LH	*p*	OR [95% CI]	RF	LF	*p*	Effect size[95% CI]
Monocular	Right	66 (97.1%)	2 (2.9%)	0.001	16.5 [3.0–91.6]	63 (92.6%)	5 (7.4%)	0.009	OR = 6.3 [1.7–24.0]
	Left	12 (66.7%)	6 (33.3%)			12 (66.7%)	6 (33.3%)		
Binocular	Right	68 (97.1%)	2 (2.9%)	< 0.001	21.6 [4.0–117.9]	66 (94.3%)	4 (5.7%)	0.001	OR = 10.5 [2.6–41.9]
	Left	11 (61.1%)	7 (38.9%)			11 (61.1%)	7 (38.9%)		

Abbreviations: LH, left‐handed; LF, left‐footed; RH, right‐handed; RF, right‐footed.

### 3.3 Stroke Characteristics

Stroke severity, assessed using the NIHSS score, was categorized as minor (NIHSS < 5, 45.9%, *n* = 45), moderate (NIHSS 5–15, 51%, *n* = 50), and severe (NIHSS > 15, 3.1%, *n* = 3). Hemiplegia was observed in 92.9% of patients, with left‐sided hemiplegia being more common (54.1%, *n* = 53) than right‐sided (38.8%, *n* = 38). Hemorrhagic strokes were localized predominantly in deep brain regions (80%, *n* = 12), while ischemic strokes were distributed across large (6.0%, *n* = 5), deep (22.9%, *n* = 19), and superficial (27.7%, *n* = 23) middle cerebral artery territories (Table 4).

**Table 4:** Stroke characteristics.

Characteristic	*N*	%
Severity (NIHSS)		
Minor (< 5)	45	45.9%
Moderate (5–15)	50	51.0%
Severe (> 15)	3	3.1%
Type of stroke		
Ischemic	83	84.7%
Hemorrhagic	15	15.3%
Hemiplegia side		
Right	38	38.8%
Left	53	54.1%
Ataxia	7	7.1%

### 3.4 Association Between Laterality and Stroke Risk Factors

**Figure 1:**
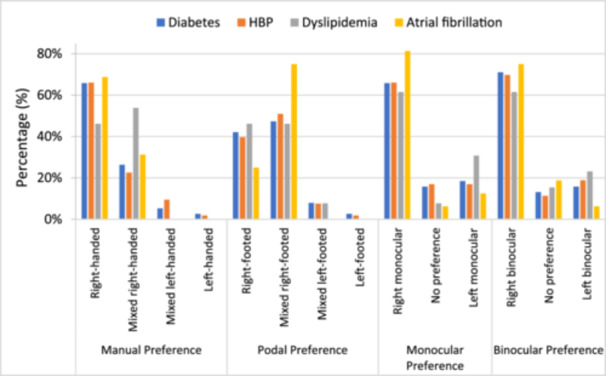
Association between laterality preferences and stroke risk factors.

Figure 1 illustrates the percentage distribution of manual, foot, and ocular laterality preferences among patients stratified by cardiovascular risk factors, namely diabetes, hypertension (HBP), dyslipidemia, and atrial fibrillation. Each stacked bar represents the comprehensive profile distribution for a specific laterality type, with color coding designating the four distinct risk factor groups. The visual representation employs a 100% stacked format to facilitate direct comparison of proportional distributions across the different laterality categories. The data reveal a consistent predominance of right‐sided laterality across all risk groups, particularly evident in manual and ocular domains, where right preferences exceed 60% in most cohorts. Notable profile variations emerge among specific comorbidities; dyslipidemia demonstrates a more balanced distribution between right and mixed manual preferences, while atrial fibrillation is characterized by a substantial proportion of mixed right‐footed preference. These descriptive findings suggest distinct patterns in laterality distribution associated with cardiovascular comorbidities, warranting further analytical investigation to elucidate potential clinical correlations. The analysis remains descriptive, with percentages derived from underlying cohort counts for each risk factor.

### 3.5 Association Between Stroke Risk Factors and Stroke Severity

Dyslipidemia was significantly associated with stroke severity (OR = 5.6, *p* = 0.033), indicating that patients with dyslipidemia were more likely to experience moderate to severe strokes. However, no significant association was found between other stroke risk factors, including diabetes, hypertension, atrial fibrillation, and stroke severity, as shown in Table 5.

**Table 5:** Association between stroke risk factors and stroke severity.

	Minor	Moderated or severe	*p*	Effect size [95% CI]
Dyslipidemia	2 (15.4%)	11 (84.6%)	0.033	OR = 5.6 [1.2–27]
No dyslipidemia	43 (50.6%)	42 (49.4%)		
Diabetes	18 (47.4%)	20 (52.6%)	0.84	OR = 0.9 [0.4–2.1]
No diabetes	27 (45%)	33 (55%)		
HBP	27 (50.9%)	26 (49.1%)	0.31	OR = 0.6 [0.3–1.4]
No HBP	18 (40%)	27 (60%)		
Atrial fibrillation	6 (37.5%)	10 (62.5%)	0.59	OR = 1.5 [0.5–4.5]
No atrial fibrillation	39 (47.6%)	43 (52.4%)		

Abbreviation: HBP, higher blood pressure.

#### 3.5.1 Association Between Laterality and Stroke Severity

Table 6 presents the association between different dimensions of laterality (manual, podal, monocular, and crossed laterality) and stroke severity (minor vs. moderate/severe). Overall, no statistically significant association was observed between any laterality variable and stroke severity (all *p* values > 0.05) in this sample.

**Table 6:** Association between stroke severity and laterality.

Laterality	Minor	Moderated or severe	*p*	Effect size [95% CI]
Manual preference				
Right‐handed + mixed right‐handed	40 (45.5%)	48 (54.5%)	> 0.99	OR = 0.8 [0.2–3.1]
Left‐handed + mixed left‐handed	5 (50%)	5 (50%)		
Manual laterality				
Right‐handed	25 (45.5%)	30 (54.5%)	> 0.99	OR = 1 [0.4–2.1]
Mixed handed	20 (46.5%)	23 (53.5%)		
Foot preference				
Right‐footed + mixed right‐footed	39 (45.3%)	47 (54.7%)	0.77	OR = 0.8 [0.2–2.8]
Left‐footed + mixed left‐footed	6 (50%)	6 (50%)		
Foot laterality				
Right‐footed	19 (48.7%)	20 (51.3%)	0.68	OR = 1.2 [0.5–2.7]
Mixed‐footed	26 (44.1%)	33 (55.9%)		
Monocular laterality				
Right monocular	36 (45%)	44 (55%)	0.80	OR = 0.8 [0.3–2.3]
Left monocular	9 (50%)	9 (50%)		
Crossed laterality hand/eye				
Uncrossed laterality	37 (44%)	47 (56%)	0.40	OR = 0.6 [0.2–1.9]
Crossed laterality	8 (57.1%)	6 (42.9%)		
Crossed laterality hand/foot/eye				
Uncrossed laterality	36 (44.4%)	45 (55.6%)	0.60	OR = 0.7 [0.2–2]
Crossed laterality	9 (52.9%)	8 (47.1%)		

Regarding manual preference, the proportion of patients with moderate/severe stroke was comparable between right‐handed or mixed right‐handed individuals (54.5%) and left‐handed or mixed left‐handed individuals (50%). The OR (OR = 0.8, 95% CI [0.2–3.1], *p* > 0.99) indicates no meaningful difference in stroke severity according to manual preference. Similarly, manual laterality (right‐handed vs. mixed‐handed) showed nearly identical distributions of severity (54.5% vs. 53.5% moderate/severe cases), with an OR of 1 (95% CI [0.4–2.1], *p* > 0.99), suggesting no association.

For foot preference, moderate/severe stroke occurred in 54.7% of right‐footed or mixed right‐footed individuals and 50% of left‐footed or mixed left‐footed individuals (OR = 0.8, 95% CI [0.2–2.8], *p* = 0.77). Foot laterality analysis also showed no significant difference between right‐footed and mixed‐footed participants (OR = 1.2, 95% CI [0.5–2.7], *p* = 0.68). Concerning monocular laterality, the distribution of stroke severity was similar between right monocular (55% moderate/severe) and left monocular participants (50% moderate/severe), with no significant association (OR = 0.8, 95% CI [0.3–2.3], *p* = 0.80). Finally, analyses of crossed laterality patterns (hand/eye and hand/foot/eye) revealed no statistically significant relationships with stroke severity. Although crossed laterality groups showed slightly different proportions of moderate/severe cases compared to uncrossed groups, the associations were not significant (hand/eye: OR = 0.6, 95% CI [0.2–1.9], *p* = 0.40; hand/foot/eye: OR = 0.7, 95% CI [0.2–2.0], *p* = 0.60). Importantly, all confidence intervals were wide and included 1, suggesting limited precision of the estimates, likely due to small subgroup sizes (particularly left‐sided and crossed laterality categories).

Spearman's rank‐order correlations revealed no significant associations between stroke severity and manual LI (*ρ* = −0.025, *p* = 0.81) or podal LI (*ρ* = 0.004, *p* = 0.97).

## 4 Discussion

This study found no significant association between laterality (handedness, footedness, or ocular dominance) and stroke severity in the sample studied. Although mixed handedness has been associated with developmental and neurological differences in certain clinical populations, such patterns were not observed in our cohort. Several studies have reported a higher prevalence of mixed handedness in specific disorders (e.g., PTSD and schizophrenia) [48]; however, these findings do not establish mixed handedness as a causal risk factor for stroke.

To date, there is no robust evidence indicating that mixed handedness constitutes an independent risk factor for stroke compared with its prevalence in the general population. Testing the hypothesis that a higher proportion of mixed handers occurs among stroke patients would require well‐controlled studies comparing stroke populations with matched control groups and employing detailed classifications of laterality (e.g., degree of dominance and multidimensional laterality profiles) [48]. Furthermore, previous research suggests that mixed handedness may be more common with increasing age, which may partly explain variability across studies.

These findings are consistent with previous research, such as Annett [49], which reported no association between laterality and poststroke severity. However, they contrast with earlier hypotheses suggesting that left‐handed individuals, due to more symmetrical brain organization, might exhibit differential stroke severity [7, 21]. The absence of an association in the present study may be partly explained by the heterogeneity of stroke subtypes (ischemic vs. hemorrhagic) and lesion locations, which may obscure potential relationship between laterality and acute stroke severity. Although left‐handed individuals may exhibit greater interhemispheric connectivity, any functional advantage related to neural plasticity may be limited to a hypothesis that warrants further investigation.

A key finding of the present study is the significant association between dyslipidemia and stroke severity. Dyslipidemia is a well‐established risk factor for atherosclerosis and stroke [50]. This observation raises the possibility that metabolic factors, rather than laterality per se, may mediate acute stroke severity. Previous studies have reported that left‐handers exhibit higher rates of cardiovascular comorbidities, including dyslipidemia, which could indirectly influence stroke severity [25, 26]. However, the relationship between laterality and metabolic health remains poorly understood and may reflect shared developmental or biological mechanisms [51]. For example, prenatal testosterone exposure has been proposed as a potential factor influencing both handedness and lipid metabolism, although current evidence remains inconclusive [52, 53].

The higher prevalence of non‐right‐handedness observed in our cohort may reflect methodological differences in handedness classification, regional cultural factors, and the stability of self‐reported hand preference even in the acute phase of stroke [54]. Although individuals with less strongly lateralized brain organization may theoretically exhibit greater neural plasticity and potentially improved recovery, this hypothesis remains speculative, as the present study did not directly assess neuroplasticity or long‐term functional outcomes. Future longitudinal studies incorporating advanced neuroimaging techniques are needed to determine whether patterns of neural reorganization differ according to laterality.

The genetic and environmental determinants of handedness represent another important area for future investigation. Twin studies and genome‐wide association studies (GWAS) have identified genetic loci associated with handedness, some of which overlap with genes implicated in cardiovascular and neurodevelopmental pathways [55, 56]. For example, the SETDB2 locus has been associated with handedness and immune‐related traits, suggesting potential shared biological mechanisms underlying lateralization and systemic health [57]. These findings suggest that shared biological mechanisms may contribute to both lateralization and vascular risk. Environmental factors, including prenatal hormonal exposure and early developmental conditions, may further influence these relationships [10, 53]. Integrating genetic, environmental, and clinical data in future studies may provide a more comprehensive understanding of how laterality relates to stroke susceptibility and outcomes.

In right‐handed individuals, patients with motor‐dominant stroke (i.e., left hemispheric lesions) have been reported to show greater responsiveness to bilateral upper‐extremity training compared with those with motor‐nondominant stroke (right‐hemispheric lesions) [58]. However, the clinical implications of such neural adaptability remain insufficiently understood. Further research examining how individuals with different laterality profiles respond to motor and language rehabilitation may clarify whether non‐right‐handed individuals exhibit distinct patterns of recovery. Such knowledge could contribute to the development of personalized rehabilitation strategies tailored to individual laterality characteristics.

### 4.1 Limitations and Future Directions

Despite its originality, this study has several limitations that must be acknowledged. First, the cross‐sectional design precludes causal inferences and limits the ability to assess long‐term recovery outcomes. Second, the underrepresentation of left‐handed individuals in the cohort may have reduced the statistical power to detect associations. Third, the study did not account for potential confounders such as socioeconomic status, educational attainment, or access to rehabilitation services, which could influence stroke outcomes. In addition, the absence of a matched control group limits the ability to determine whether the observed distribution of laterality differs from that of the general population.

Future research should address these limitations through longitudinal study designs, larger, balanced and more diverse cohorts, and the integration of genetic and neuroimaging data. Although the present findings reflect patterns observed in a Tunisian stroke population, cultural and regional differences in laterality prevalence, such as higher reported rates of left‐handedness in Western populations, may influence generalizability. Multicenter studies across diverse populations are therefore needed to clarify potential population‐specific differences.

### 4.2 Clinical and Research Implications

Despite these limitations, the present findings have several implications for clinical practice and future research. Clinically, the observed association between dyslipidemia and stroke severity underscores the need for rigorous management of metabolic risk factors in patients with stroke, particularly those who are left‐handed. This finding suggests that metabolic factors may contribute more directly to acute stroke severity than laterality itself. From a research perspective, the study highlights the need for further investigation into the relationships among laterality, neuroplasticity, and metabolic health. Studies conducted in larger and genetically diverse populations may help clarify whether shared biological mechanisms underlie these associations. A better understanding of these relationships may contribute to the development of individualized approaches to stroke prevention and rehabilitation.

## 5 Conclusions

The present study explored the association between laterality (specifically, handedness, footedness, and ocular preference) and stroke severity in a cohort of 98 patients with ischemic or hemorrhagic stroke. While the findings did not reveal a significant correlation between laterality and stroke severity, they underscored the complexity of brain lateralization and its potential implications for neurological outcomes. The absence of a direct association may be attributed to the limited sample size of left‐handed and left‐footed individuals, as well as the multifactorial nature of stroke pathophysiology, which involves genetic, environmental, and lifestyle factors. The study confirmed a strong correlation between manual and foot preferences, aligning with existing literature on the interconnectedness of lateralized behaviors. However, the lack of a significant link between laterality and stroke severity suggests that other factors (dyslipidemia and lesion location) may play more important roles in determining acute stroke severity. Results suggest that dyslipidemia is a significant predictor of stroke severity, consistent with prior research linking handedness to cardiovascular risk factors. These findings highlight the need for further investigation into the genetic and environmental underpinnings of laterality and their potential influence on stroke risk and recovery. While dyslipidemia was significantly associated with stroke severity, the small subgroup of left‐handed individuals precludes a definitive conclusion about a direct link between handedness and dyslipidemia. The observed trend warrants investigation in larger, specifically powered studies. Larger and more diverse cohorts, including a greater representation of left‐handed and left‐footed individuals, are essential to further explain these relationships. Additionally, integrating genetic analyses, such as GWAS, could unveil the biological mechanisms linking laterality to stroke outcomes.

## Author Contributions

Conceptualization: Imen Ezzouch, Riadh Dahmen, and Mohamed Jarraya. Methodology: Imen Ezzouch, Riadh Dahmen, Achraf Ammar, Haitham Jahrami, and Mohamed Jarraya. Software: Liwa Masmoudi and Imen Ezzouch. Validation: Riadh Dahmen, Saoussen Daoued, Achraf Ammar, Haitham Jahrami, Khaled Trabelsi, and Mohamed Jarraya. Formal analysis: Imen Ezzouch and Liwa Masmoudi. Investigation: Imen Ezzouch, Saoussen Daoued, Sana Ben Amor, Mariem Dammak, and Chokri Mhiri. Writing – original draft preparation: Imen Ezzouch. Writing – review and editing: Imen Ezzouch, Kaouther Mejri, Achraf Ammar, Dalia Elleuch, Haitham Jahrami, Saoussen Daoued, and Khaled Trabelsi. Supervision: Riadh Dahmen, Achraf Ammar, Khaled Trabelsi, Haitham Jahrami, and Mohamed Jarraya. Resources: Achraf Ammar, Mariem Dammak, and Chokri Mhiri. All authors have read and approved the final version of the manuscript.

## Funding

The authors received no specific funding for this work.

## Ethics Statement

Ethical approval was obtained from the Habib Bourguiba University Hospital Ethics Committee (Reference No. 452/2022).

## Consent

Informed consent was obtained from all subjects involved in the study.

## Conflicts of Interest

The authors declare no conflicts of interest.

## Transparency Statement

The lead author Imen Ezzouch affirms that this manuscript is an honest, accurate, and transparent account of the study being reported; that no important aspects of the study have been omitted; and that any discrepancies from the study as planned (and, if relevant, registered) have been explained.

## Supporting information

Supporting File S1_STROBE_Checklist.

## Data Availability

Deidentified participant data are available upon reasonable request from the first author, subject to ethical approvals. Imen Ezzouch had full access to all of the data in this study and takes complete responsibility for the integrity of the data and the accuracy of the data analysis.
