# Supplementary material for: The Association Between Laterality and Stroke Severity: A Cross‐Sectional Study
Source: Health Sci Rep. 2026 Mar 22;9(3):e72102. doi: 10.1002/hsr2.72102 (PMC13098056; doi:10.1002/hsr2.72102)
Supplement: Supplementary file 1 — Supporting File S1_STROBE_Checklist. [file HSR2-9-e72102-s001.docx]

STROBE Checklist of items that should be included in reports of ***cross-sectional studies***

|  | Item No | Recommendation | Page No |
| --- | --- | --- | --- |
| **Title and abstract** | 1 | (*a*) Indicate the study’s design with a commonly used term in the title or the abstract | Title: "The Influence of Laterality on Stroke Severity: A Cross-Sectional Study" (p. 1) |
|  |  | (*b*) Provide in the abstract an informative and balanced summary of what was done and what was found | \| Abstract (p. 1) \| \| --- \| |
| Introduction | | | |
| Background/rationale | 2 | Explain the scientific background and rationale for the investigation being reported | \| Introduction (pp. 1–2) \| \| --- \| |
| Objectives | 3 | State specific objectives, including any prespecified hypotheses | \| Abstract (Objective) & Introduction (final paragraph) \| \| --- \| |
| Methods | | | |
| Study design | 4 | Present key elements of study design early in the paper | \| Methods (Study Design) \| \| --- \|   (p. 4) |
| Setting | 5 | Describe the setting, locations, and relevant dates, including periods of recruitment, exposure, follow-up, and data collection | Methods (Participants: Habib Bourguiba/Sahloul Hospitals, Tunisia) (p. 4) |
| Participants | 6 | (*a*) Give the eligibility criteria, and the sources and methods of selection of participants | \| Methods (Inclusion/Exclusion Criteria) (p. 4) \| \| --- \| |
| Variables | 7 | Clearly define all outcomes, exposures, predictors, potential confounders, and effect modifiers. Give diagnostic criteria, if applicable | \| Methods (Laterality, NIHSS, risk factors) (p. 4) \| \| --- \| |
| Data sources/ measurement | 8* | For each variable of interest, give sources of data and details of methods of assessment (measurement). Describe comparability of assessment methods if there is more than one group | \| Methods (NIHSS, Dellatolas/Dahmen inventories) \| \| --- \|   (p. 4) |
| Bias | 9 | Describe any efforts to address potential sources of bias | \| Bias considerations (p. 6) \| \| --- \| |
| Study size | 10 | Explain how the study size was arrived at | \| Methods (Study Design) \| \| --- \|   (p. 4) |
| Quantitative variables | 11 | Explain how quantitative variables were handled in the analyses. If applicable, describe which groupings were chosen and why | \| Methods (Study Design) \| \| --- \|   (p. 4) |
| Statistical methods | 12 | (*a*) Describe all statistical methods, including those used to control for confounding | \| Methods (Laterality Index formula) (p. 6) \| \| --- \| |
|  |  | (*b*) Describe any methods used to examine subgroups and interactions | \| Methods (Mann-Whitney U, Kruskal-Wallis) (p. 6) \| \| --- \| |
|  |  | (*c*) Explain how missing data were addressed | Methods (p. 6) |
|  |  | (*d*) If applicable, describe analytical methods taking account of sampling strategy | N/A |
|  |  | (*e*) Describe any sensitivity analyses | N/A |
| Results | | | |
| Participants | 13* | (a) Report numbers of individuals at each stage of study—eg numbers potentially eligible, examined for eligibility, confirmed eligible, included in the study, completing follow-up, and analysed | Results (p. 7) |
|  |  | (b) Give reasons for non-participation at each stage | Exclusion Criteria (p. 4) |
|  |  | (c) Consider use of a flow diagram | N/A |
| Descriptive data | 14* | (a) Give characteristics of study participants (eg demographic, clinical, social) and information on exposures and potential confounders | Table 1 (p. 17) |
|  |  | (b) Indicate number of participants with missing data for each variable of interest | N/A |
| Outcome data | 15* | Report numbers of outcome events or summary measures | Results (p. 6) |
| Main results | 16 | (*a*) Give unadjusted estimates and, if applicable, confounder-adjusted estimates and their precision (eg, 95% confidence interval). Make clear which confounders were adjusted for and why they were included | Results (p. 6-7) |
|  |  | (*b*) Report category boundaries when continuous variables were categorized | Results (p. 6-8) |
|  |  | (*c*) If relevant, consider translating estimates of relative risk into absolute risk for a meaningful time period | N/A |
| Other analyses | 17 | Report other analyses done—eg analyses of subgroups and interactions, and sensitivity analyses | Results (p. 6-8) |
| Discussion | | | |
| Key results | 18 | Summarise key results with reference to study objectives | Results (p. 8) |
| Limitations | 19 | Discuss limitations of the study, taking into account sources of potential bias or imprecision. Discuss both direction and magnitude of any potential bias | Discussion (pp. 8-9) |
| Interpretation | 20 | Give a cautious overall interpretation of results considering objectives, limitations, multiplicity of analyses, results from similar studies, and other relevant evidence | Discussion (pp. 8-9) |
| Generalisability | 21 | Discuss the generalisability (external validity) of the study results | Discussion (pp. 8-9) |
| Other information | | | |
| Funding | 22 | Give the source of funding and the role of the funders for the present study and, if applicable, for the original study on which the present article is based | No funding (p. 12) |
